# Supplementary material for: Immunomodulatory Effects of IFNα on T and NK Cells in Chronic Myeloid Leukemia Patients in Deep Molecular Response Preparing for Treatment Discontinuation
Source: J Clin Med. 2022 Sep 23;11(19):5594. doi: 10.3390/jcm11195594 (PMC9570842; doi:10.3390/jcm11195594)
Supplement: Supplementary file 1 [file jcm-11-05594-s001.zip › 813834_Table_1 (2).pdf]

Table S1. Lymphocyte subpopulations: (A) percent and (B) absolute count.

A

			T cells	CD4$^+$ T cells	CD8$^+$ T cells	NKT cells	NK cells	CD56^{bright} CD16$^-$	CD56^{bright} CD16^{dim}	CD56^{dim} CD16$^+$
IFNα-only	Median		**63.9%**	**43.2%**	**23.6%**	**5.1%**	**11.0%**	**2.6%**	**3.5%**	**92.6%**
	Interquartile range	25	55.1%	32.2%	19.1%	1.6%	9.3%	1.5%	3.0%	87.5%
		75	77.2%	48.9%	29.1%	7.9%	22.6%	4.1%	6.7%	95.1%
IFNα+TKI	Median		**57.4%**	**39.7%**	**17.7%**	**3.6%**	**14.3%**	**3.0%**	**2.7%**	**94.6%**
	Interquartile range	25	32.6%	23.1%	10.6%	2.0%	7.1%	1.7%	1.9%	92.2%
		75	64.7%	47.1%	19.3%	9.3%	16.9%	4.8%	4.0%	94.9%
TKI-only	Median		**60.1%**	**41.5%**	**13.6%**	**5.0%**	**15.6%**	**2.8%**	**3.9%**	**91.2%**
	Interquartile range	25	44.0%	24.3%	10.6%	3.3%	10.8%	1.9%	1.8%	87.5%
		75	67.2%	52.2%	19.7%	8.9%	20.6%	4.9%	6.5%	95.7%
Overall	Median		**60.1%**	**41.5%**	**17.4%**	**4.7%**	**14.2%**	**2.8%**	**3.4%**	**92.8%**
	Interquartile range	25	47.6%	28.8%	10.9%	3.1%	10.3%	1.9%	1.9%	89.5%
		75	67.2%	49.0%	21.5%	8.7%	19.1%	4.7%	6.3%	95.0%

B

			T cells	CD4$^+$ T cells	CD8$^+$ T cells	NKT cells	NK cells	CD56^{bright} CD16$^-$	CD56^{bright} CD16^{dim}	CD56^{dim} CD16$^+$
IFNα-only	Median		**1425**	**862**	**519**	**109**	**310**	**7**	**13**	**289**
	Interquartile range	1161	786	340	28	136	5	7	120	87.5%
		1739	976	664	214	564	10	20	535	95.1%
IFNα+TKI	Median		**981**	**593**	**331**	**60**	**186**	**8**	**6**	**175**
	Interquartile range	528	382	138	28	114	3	3	108	92.2%
		1313	981	391	200	342	11	14	306	94.9%
TKI-only	Median		**977**	**628**	**238**	**67**	**236**	**5**	**7**	**217**
	Interquartile range	610	386	154	46	139	4	4	122	87.5%
		1280	985	390	181	387	10	18	367	95.7%
Overall	Median		**1114**	**678**	**285**	**73**	**250**	**6**	**8**	**228**
	Interquartile range	25	620	436	153	40	138	4	5	122
		75	1372	976	408	181	387	10	14	364
