# Supplementary material for: Immunomodulatory Effects of IFNα on T and NK Cells in Chronic Myeloid Leukemia Patients in Deep Molecular Response Preparing for Treatment Discontinuation
Source: J Clin Med. 2022 Sep 23;11(19):5594. doi: 10.3390/jcm11195594 (PMC9570842; doi:10.3390/jcm11195594)
Supplement: Supplementary file 1 [file jcm-11-05594-s001.zip › 813834_Table_2.pdf]

Table S2. Percentage of cytokine producing cells

			Percentage of IFNγ producing cells						Percentage of TNFα producing cells						T-cell cytokine production		
			$CD4^+$ T cells	$CD8^+$ T cells	NK cells	NK $CD56^{bright}/CD16^-$	NK $CD56^{dim}/CD16^+$	NKT cells	$CD4^+$ T cells	$CD8^+$ T cells	NK cells	NK $CD56^{bright}/CD16^-$	NK $CD56^{dim}/CD16^+$	NKT cells	$CD3^+CD4^+IFN\gamma^+$ cells (Th1)	$CD3^+CD4^+IL\text{-}4^+$ cells (Th2)	$CD3^+CD4^+IL\text{-}17A^+$ cells (Th17)
IFNα-only	**Median**		**27.5%**	**70.2%**	**82.1%**	**91.9%**	**83.2%**	**98.7%**	**80.8%**	**77.1%**	**56.7%**	**68.1%**	**56.1%**	**92.7%**	**29.7%**	**5.3%**	**2.3%**
	**Interquartile range**	**25**	18.9%	43.9%	72.8%	88.5%	71.6%	92.0%	66.3%	64.3%	36.9%	56.1%	36.2%	69.8%	24.0%	4.5%	1.7%
		**75**	33.8%	79.8%	84.5%	94.3%	83.7%	99.6%	84.0%	84.8%	68.2%	75.3%	78.5%	95.6%	38.4%	9.5%	3.4%
IFNα+TKI	**Median**		**34.5%**	**77.3%**	**89.5%**	**90.9%**	**88.8%**	**95.8%**	**73.4%**	**74.3%**	**45.0%**	**46.3%**	**41.7%**	**90.2%**	**35.7%**	**4.8%**	**1.9%**
	**Interquartile range**	**25**	23.7%	61.6%	68.2%	82.6%	62.6%	86.0%	61.9%	59.8%	36.7%	29.3%	36.3%	73.7%	21.6%	2.8%	1.0%
		**75**	45.2%	88.1%	95.0%	94.1%	93.7%	97.6%	77.8%	86.1%	52.3%	54.3%	53.5%	99.7%	46.9%	9.2%	3.4%
TKI-only	**Median**		**21.7%**	**58.8%**	**80.9%**	**90.2%**	**76.7%**	**82.1%**	**63.7%**	**62.2%**	**51.7%**	**49.2%**	**50.7%**	**87.2%**	**18.9%**	**2.6%**	**1.6%**
	**Interquartile range**	**25**	14.9%	45.1%	68.1%	78.2%	53.4%	64.5%	42.3%	43.3%	40.8%	37.3%	37.6%	71.0%	15.8%	1.9%	1.3%
		**75**	29.8%	80.8%	90.5%	93.2%	84.3%	92.9%	78.7%	84.7%	62.1%	63.2%	64.8%	91.5%	28.0%	4.2%	2.3%
Overall	**Median**		**24.5%**	**67.8%**	**82.1%**	**91.0%**	**76.9%**	**89.8%**	**72.6%**	**71.4%**	**50.2%**	**51.4%**	**50.7%**	**89.3%**	**26.4%**	**3.9%**	**2.0%**
	**Interquartile range**	**25**	16.4%	53.1%	69.9%	82.9%	64.4%	69.0%	59.0%	53.4%	38.0%	37.3%	36.7%	72.2%	17.9%	2.4%	1.3%
		**75**	34.4%	80.9%	90.5%	93.5%	88.9%	97.1%	80.8%	84.7%	62.1%	67.0%	64.8%	93.8%	34.0%	7.0%	2.8%
