# Supplementary material for: Immunomodulatory Effects of IFNα on T and NK Cells in Chronic Myeloid Leukemia Patients in Deep Molecular Response Preparing for Treatment Discontinuation
Source: J Clin Med. 2022 Sep 23;11(19):5594. doi: 10.3390/jcm11195594 (PMC9570842; doi:10.3390/jcm11195594)
Supplement: Supplementary file 1 [file jcm-11-05594-s001.zip › 813834_Table_3.pdf]

Table S3. Cytokine production. Number of cytokine-producing cells/µl

			IFNγ production/µl						TNFα production/µl						T-cell cytokine production		
			CD4^{+} T cells	CD8^{+} T cells	NK cells	NK CD56^{bright}/CD16^{-}	NK CD56^{dim}/CD16^{+}	NKT cells	CD4^{+} T cells	CD8^{+} T cells	NK cells	NK CD56^{bright}/CD16^{-}	NK CD56^{dim}/CD16^{+}	NKT cells	CD3^{+}CD4^{+}IFNγ^{+} cells (Th1)	CD3^{+}CD4^{+}IL-4^{+} cells (Th2)	CD3^{+}CD4^{+}IL-17A^{+} cells (Th17)
IFNα-only	Median		**197**	**234**	**259**	**10**	**189**	**122**	**616**	**289**	**159**	**8**	**111**	**96**	**258.1**	**49.61**	**17.7**
	Interquartile range	25	142	143	108	6	99	58	457	160	75	3	66	44	202.0	41.68	15.5
		75	318	492	372	18	252	286	860	535	314	14	166	276	333.3	95.49	32.2
IFNα+TKI	Median		**169**	**207**	**164**	**7**	**155**	**45**	**499**	**208**	**114**	**3**	**114**	**48**	**303.5**	**35.62**	**15.5**
	Interquartile range	25	102	96	61	3	56	21	227	92	55	1	53	20	144.7	22.91	7.0
		75	369	273	274	10	254	136	771	285	152	5	145	103	405.0	52.34	21.2
TKI-only	Median		**132**	**152**	**185**	**5**	**153**	**54**	**385**	**141**	**97**	**3**	**86**	**53**	**177.5**	**21.93**	**16.6**
	Interquartile range	25	77	108	78	2	70	23	184	91	45	2	39	22	108.0	12.87	5.0
		75	173	230	319	9	305	154	630	193	235	6	242	138	230.7	27.95	20.8
Overall	Median		**156**	**168**	**193**	**6**	**159**	**65**	**460**	**166**	**111**	**4**	**105**	**62**	**197.6**	**27.38**	**17.0**
	Interquartile range	25	102	117	78	3	70	28	250	95	55	2	53	27	139.5	19.88	7.7
		75	239	262	317	10	254	140	686	259	194	7	174	128	294.8	45.62	21.7
