# Supplementary material for: Immunomodulatory Effects of IFNα on T and NK Cells in Chronic Myeloid Leukemia Patients in Deep Molecular Response Preparing for Treatment Discontinuation
Source: J Clin Med. 2022 Sep 23;11(19):5594. doi: 10.3390/jcm11195594 (PMC9570842; doi:10.3390/jcm11195594)
Supplement: Supplementary file 1 [file jcm-11-05594-s001.zip › 813834_Table_4.pdf]

Table S4. Maturation stage, activation markers and activating receptors on NK subpopulations

			Percentage of NKG2D				Percentage of NKp30				Percentage of NKp44				Percentage of NKp46			
			**NK cells**	**CD56^{bright}/CD16^{-} NK cells**	**CD56^{bright}/CD16^{dim} NK cells**	**CD56^{dim}/CD16^{+} NK cells**	**NK cells**	**CD56^{bright}/CD16^{-} NK cells**	**CD56^{bright}/CD16^{dim} NK cells**	**CD56^{dim}/CD16^{+} NK cells**	**NK cells**	**CD56^{bright}/CD16^{-} NK cells**	**CD56^{bright}/CD16^{dim} NK cells**	**CD56^{dim}/CD16^{+} NK cells**	**NK cells**	**CD56^{bright}/CD16^{-} NK cells**	**CD56^{bright}/CD16^{dim} NK cells**	**CD56^{dim}/CD16^{+} NK cells**
IFNα-only	**Median**		**93.9%**	**94.3%**	**98.2%**	**94.4%**	**9.4%**	**16.5%**	**19.4%**	**8.9%**	**5.3%**	**34.9%**	**23.1%**	**4.3%**	**31.5%**	**76.8%**	**68.2%**	**28.8%**
	**Interquartile range**	**25**	83.5%	93.0%	95.4%	87.6%	6.5%	6.2%	9.4%	6.7%	3.4%	18.4%	13.5%	1.8%	13.9%	28.3%	34.4%	13.1%
		**75**	97.4%	98.1%	98.5%	98.9%	19.3%	24.3%	31.2%	18.3%	15.5%	43.7%	39.8%	10.4%	56.0%	92.4%	76.9%	56.5%
IFNα+TKI	**Median**		**95.5%**	**92.5%**	**99.7%**	**97.8%**	**11.8%**	**16.9%**	**7.5%**	**11.9%**	**3.5%**	**17.9%**	**14.3%**	**2.7%**	**37.7%**	**69.7%**	**71.7%**	**34.2%**
	**Interquartile range**	**25**	92.8%	86.0%	93.7%	95.1%	3.7%	4.1%	3.3%	3.1%	1.4%	6.6%	6.7%	0.5%	22.5%	52.5%	57.8%	22.4%
		**75**	99.2%	98.1%	100.0%	99.5%	56.4%	65.3%	23.1%	54.5%	6.8%	25.8%	15.4%	5.4%	67.7%	77.6%	93.0%	69.1%
TKI-only	**Median**		**95.7%**	**92.1%**	**99.2%**	**98.3%**	**20.9%**	**19.5%**	**15.7%**	**15.4%**	**4.0%**	**27.0%**	**13.6%**	**1.9%**	**37.5%**	**73.8%**	**70.2%**	**34.4%**
	**Interquartile range**	**25**	93.5%	74.7%	90.2%	95.0%	3.6%	4.3%	7.2%	4.2%	1.8%	6.5%	8.4%	0.7%	13.8%	43.3%	52.1%	13.6%
		**75**	98.5%	97.1%	100.0%	99.7%	61.5%	52.8%	44.9%	59.7%	10.2%	55.3%	36.8%	5.3%	69.0%	90.4%	91.1%	71.1%
Overall	**Median**		**95.6%**	**93.6%**	**98.5%**	**98.0%**	**12.4%**	**16.8%**	**14.4%**	**12.5%**	**4.4%**	**25.0%**	**14.9%**	**2.6%**	**35.5%**	**71.7%**	**69.8%**	**29.0%**
	**Interquartile range**	**25**	92.9%	83.2%	94.8%	94.3%	5.9%	4.9%	7.3%	5.4%	1.8%	7.5%	8.7%	0.8%	17.9%	45.1%	52.7%	15.3%
		**75**	98.3%	97.4%	100.0%	99.5%	54.0%	45.8%	31.7%	51.5%	8.1%	47.4%	33.1%	5.5%	66.3%	89.8%	90.8%	66.9%
