# Supplementary material for: Immunomodulatory Effects of IFNα on T and NK Cells in Chronic Myeloid Leukemia Patients in Deep Molecular Response Preparing for Treatment Discontinuation
Source: J Clin Med. 2022 Sep 23;11(19):5594. doi: 10.3390/jcm11195594 (PMC9570842; doi:10.3390/jcm11195594)
Supplement: Supplementary file 1 [file jcm-11-05594-s001.zip › 813834_Table_5.pdf]

Table S5. Cytotoxic activity of NK cells against the K562 cancer cell line. Data are expressed as median percentage of lysis

		IFNα-only				IFNα+TKI				TKI-only			
E:T ratio		100:1	50:1	25:1	12.5:1	100:1	50:1	25:1	12.5:1	100:1	50:1	25:1	12.5:1
Median		53.9%	37.7%	16.2%	23.1%	50.5%	42.9%	26.7%	16.0%	33.6%	33.9%	19.0%	12.3%
Interquartile range	25	40.2%	20.2%	5.2%	3.6%	28.3%	20.5%	12.0%	6.3%	16.5%	10.6%	7.6%	4.1%
	75	67.7%	56.8%	40.2%	25.9%	57.8%	60.0%	38.3%	20.6%	60.3%	51.0%	47.3%	34.9%
